# Influences on Skin and Intrinsic Aging: Biological, Environmental, and Therapeutic Insights

**DOI:** 10.1111/jocd.16688

**Published:** 2024-11-27

**Authors:** Ramadan S. Hussein, Salman Bin Dayel, Othman Abahussein, Abeer Ali El‐Sherbiny

**Affiliations:** ^1^ Dermatology Unit, Department of Internal Medicine, College of Medicine Prince Sattam Bin Abdulaziz University Al‐Kharj Saudi Arabia; ^2^ Department of Medical Laboratory, College of Applied Medical Sciences Prince Sattam bin Abdulaziz University Al‐Kharj Saudi Arabia

**Keywords:** biological mechanisms, environmental factors, extrinsic aging, intrinsic aging, skin aging

## Abstract

**Background/Aim:**

Aging involves a progressive deterioration in physiological functions and increased disease susceptibility, impacting all organs and tissues, especially the skin. Skin aging is driven by intrinsic factors (genetics, cellular metabolism) and extrinsic factors (environment, lifestyle). Understanding these mechanisms is vital for promoting healthy aging and mitigating skin aging effects. This review aims to summarize the key factors influencing skin and intrinsic aging, providing a comprehensive understanding of the underlying mechanisms and contributing elements.

**Methods:**

A comprehensive literature review was conducted, focusing on peer‐reviewed journals, clinical studies, and scientific reviews published within the last two decades. The inclusion criteria prioritized studies that addressed intrinsic and extrinsic mechanisms of skin aging. To ensure the relevance and quality of the selected sources, a systematic approach was used to assess study design, sample size, methodology, and the significance of the findings in the context of skin aging.

**Findings:**

The review identifies major internal factors, such as cellular senescence, genetic predisposition, telomere shortening, oxidative stress, hormonal changes, metabolic processes, and immune system decline, as pivotal contributors to intrinsic aging. External factors, including UV radiation, pollution, lifestyle choices (diet, smoking, alcohol consumption, and sleep patterns), and skincare practices, significantly influence extrinsic skin aging. The interplay between these factors accelerates aging processes, leading to various clinical manifestations like wrinkles, loss of skin elasticity, pigmentation changes, and texture alterations.

**Conclusion:**

A comprehensive understanding of both extrinsic and intrinsic factors contributing to skin aging is essential for developing effective prevention and intervention strategies. The insights gained from this review highlight the importance of a multifaceted approach, incorporating lifestyle modifications, advanced skincare routines, and emerging therapeutic technologies, to mitigate the effects of aging and promote healthier, more resilient skin.

## 1 Introduction

Aging is a multifaceted biological process marked by a progressive decline in physiological functions and an increased vulnerability to diseases. It affects all organs and tissues, with the skin serving as one of the most evident indicators of aging [1]. Skin aging is influenced by intrinsic factors like genetics and cellular metabolism, as well as extrinsic factors such as environmental exposures and lifestyle choices [2, 3]. Understanding these mechanisms is crucial for developing strategies that encourage healthy aging and mitigate the negative impacts on skin appearance and function.

The relevance of studying skin and intrinsic aging extends beyond cosmetic concerns. The skin serves as the body's primary barrier against environmental insults and plays a critical role in overall health [4]. As such, insights into the factors that drive skin aging can inform broader health strategies and interventions [5]. In the realm of cosmetic science, comprehending the intricacies of skin aging is essential for innovating products and treatments that effectively combat the visible signs of aging, thereby improving the quality of life and boosting self‐esteem [6].

In addition to the biological mechanisms of skin aging, the economic and societal relevance of the aging and anti‐aging market has grown significantly. The global anti‐aging market, valued at over $60 billion, reflects a substantial and increasing demand for products and treatments that target aging [7]. Middle‐aged and older adults have historically been the primary consumers of anti‐aging products, but recent trends indicate a growing interest from younger demographics seeking preventative measures [8, 9]. Shifts in public opinion also reflect a broader acceptance of anti‐aging treatments, with many consumers focusing on holistic approaches that integrate skincare, lifestyle adjustments, and emerging therapeutic technologies [9].

This review aims to summarize the biological basis of aging, focusing on cellular senescence, genetic factors, telomere shortening, and oxidative stress. It identifies and discusses intrinsic factors influencing skin aging, including hormonal changes, metabolic processes, immune system decline, and genetic predisposition. Additionally, it examines extrinsic factors affecting skin aging, such as UV radiation, pollution, lifestyle choices, and skincare practices. The review explores the molecular mechanisms underpinning skin aging, including collagen and elastin breakdown, matrix metalloproteinases (MMPs), glycation, and chronic inflammation. Furthermore, it details the clinical manifestations of aging skin, including wrinkles, loss of firmness, pigmentation changes, and alterations in texture. Current methods for assessing and measuring skin aging are reviewed, and various interventions and prevention strategies, ranging from topical treatments to lifestyle modifications and emerging therapies, are discussed. Finally, the review highlights future directions and identifies gaps in the current research landscape to encourage further investigation in the field [4, 5, 10, 11].

### 1.1 Methods

A comprehensive literature search was conducted to identify relevant studies on skin aging and intrinsic aging mechanisms. The databases used for the search included PubMed, Web of Science, and Scopus. The search focused on peer‐reviewed articles, clinical studies, and scientific reviews published in the last two decades (2004–2024).

Search terms included combinations of keywords such as “skin aging,” “intrinsic aging,” “extrinsic aging,” “cellular senescence,” “genetic factors,” “oxidative stress,” “hormonal changes,” “UV radiation,” “pollution,” and “lifestyle factors.” Boolean operators (AND, OR) were used to refine the search, and articles were filtered based on relevance to the review's focus on biological mechanisms, clinical manifestations, and therapeutic interventions for skin aging.

Studies were selected based on the inclusion criteria of being peer‐reviewed publications, addressing intrinsic and extrinsic factors influencing skin aging, published in English, and providing mechanistic insights or clinical data related to aging interventions, while exclusion criteria consisted of non‐peer‐reviewed sources (e.g., conference abstracts, opinion pieces), studies not directly related to skin aging or intrinsic aging mechanisms, and research with insufficient methodological details or unclear findings.

Two independent reviewers screened the abstracts and full texts of the selected studies to ensure eligibility. Any discrepancies in study selection were resolved through discussion. For quality assessment, each study was evaluated based on its design, sample size, methodology, and relevance to skin aging. Only studies meeting a high standard of scientific rigor were included in the final review.

The literature search was performed between January and March 2024 to capture the most recent research and developments in the field of skin aging.

## 2 Biological Basis of Aging

### 2.1 Cellular Senescence

Cellular senescence denotes the permanent halt in cell division triggered by either cellular stress or reaching a critical number of divisions. It acts as a protective mechanism to prevent the proliferation of damaged cells, thus reducing the risk of cancer. However, as individuals age, senescent cells accumulate and play a role in aging through the secretion of inflammatory cytokines, proteases, and growth factors, collectively termed the senescence‐associated secretory phenotype (SASP) [12].

In the skin, cellular senescence disrupts tissue homeostasis and impairs the regenerative capacity of skin cells. Accumulation of senescent fibroblasts leads to a decreased production of collagen and elastin, critical components of the skin's extracellular matrix. As a consequence, this leads to diminished skin elasticity, heightened formation of wrinkles, and delayed wound healing. Senescent keratinocytes and melanocytes also contribute to age‐related alterations in skin texture and pigmentation [13].

### 2.2 Genetic Factors

Genetic factors exert considerable influence in determining the rate and manner of aging. Variations in genes involved in DNA repair, antioxidant defense, and metabolic processes can influence longevity and susceptibility to age‐related diseases. For instance, mutations in the Werner syndrome ATP‐dependent helicase (WRN) gene, responsible for Werner syndrome, accelerate aging and manifest as premature skin aging [14].

The genetic predisposition affects intrinsic aging by determining baseline cellular functions and responses to environmental stressors. Genetic variants in the matrix metalloproteinase 1 (MMP1) gene can influence the rate of collagen breakdown in the skin, affecting wrinkle formation. Polymorphisms in genes associated with oxidative stress responses, such as superoxide dismutase 2 (SOD2) and glutathione peroxidase 1(GPX1), can modify an individual's ability to neutralize free radicals, impacting skin aging [6].

### 2.3 Telomere Shortening

Telomeres are repetitive sequences of nucleotides located at the ends of chromosomes, serving to protect them from deterioration or fusion with adjacent chromosomes. Each time a cell divides, its telomeres shorten due to the end‐replication problem. When telomeres reach a critically short length, cells either enter a state of senescence or undergo apoptosis [15].

Telomere shortening is a hallmark of cellular aging. It limits the replicative potential of cells, contributing to the decline in tissue function over time. In the skin, telomere shortening affects proliferative cells such as keratinocytes and fibroblasts, leading to reduced skin regeneration and repair capacity. This process is exacerbated by oxidative stress and chronic inflammation, further accelerating skin aging [16].

### 2.4 Oxidative Stress and Free Radicals

Oxidative stress arises when there is an imbalance between the production of reactive oxygen species (ROS) and the skin's capacity to detoxify these reactive molecules or repair the damage they cause. Sources of ROS include mitochondrial respiration, UV radiation, pollution, and inflammatory processes [17].

Free radicals, including hydrogen peroxide, superoxide anions, and hydroxyl radicals, can damage cellular components such as lipids, proteins, and DNA. In the skin, oxidative stress causes lipid peroxidation in cell membranes, protein oxidation, and DNA strand breaks [18]. These damages impair cellular function and accelerate the aging process. Specifically, oxidative stress degrades collagen and elastin, leading to the loss of skin firmness and elasticity. It also triggers inflammatory pathways and increases the expression of matrix metalloproteinases (MMPs), enzymes that break down collagen, further contributing to wrinkle formation and skin thinning [19, 20].

## 3 Intrinsic Factors Influencing Skin Aging

### 3.1 Hormonal Changes

Menopause brings significant hormonal changes, particularly a decline in estrogen levels, which profoundly affect skin aging. Estrogen is essential for maintaining the skin's thickness, hydration, and elasticity. Its decline leads to a decreased collagen production, a reduced skin moisture, and an increased wrinkle formation. Post‐menopausal women often experience accelerated skin aging, characterized by dryness, thinning, and loss of elasticity [10, 21].

In men, declining testosterone levels with age also impact skin health. Testosterone helps maintain skin thickness and oil production. Its reduction can lead to drier skin and a decrease in skin elasticity. Although the effects are generally less pronounced than those associated with estrogen decline, they still contribute to the overall aging process in the skin [10].

Other hormones, including growth hormone and dehydroepiandrosterone (DHEA), also influence skin aging. Growth hormone helps stimulate cell regeneration and repair, while DHEA is involved in the production of sebum, contributing to skin hydration and barrier function. Declines in these hormones with age can further exacerbate the signs of skin aging [22].

### 3.2 Metabolic Processes

Metabolism affects skin aging through the regulation of cellular energy production, nutrient supply, and waste removal. As metabolic processes slow down with age, cells receive less energy and nutrients, which impairs their function and regeneration capacity. Reduced metabolic activity also means slower removal of cellular waste, contributing to the buildup of damaged proteins and lipids in the skin [20, 23].

Metabolic processes can produce advanced glycation end products (AGEs), which are proteins or lipids that become glycated due to exposure to sugars. AGEs can cross‐link collagen fibers, making them stiff and less elastic, thereby contributing to wrinkles and reduced skin suppleness [23, 24].

### 3.3 Immune System Decline

The immune system is essential for maintaining skin health by protecting against pathogens, facilitating wound healing, and modulating inflammation. With age, the immune system becomes less effective, a phenomenon known as immunosenescence. This decline results in reduced skin resilience, slower healing of wounds, and increased susceptibility to infections and skin diseases. The decline in immune function with age is characterized by a decrease in the number and functionality of immune cells, including T‐cells and macrophages, which impairs the skin's ability to respond to insults and repair itself effectively [25].

In the context of aging, specific cytokines play a significant role in inflammation and its impact on skin health. Pro‐inflammatory cytokines such as interleukin‐6 (IL‐6), tumor necrosis factor‐alpha (TNF‐α), and interleukin‐1 beta (IL‐1β) are pivotal in this process. IL‐6 is known for its role in chronic inflammation and has been associated with reduced skin elasticity and increased susceptibility to skin disorders in the elderly. TNF‐α contributes to the inflammatory cascade and has been linked to age‐related skin thinning and impaired wound healing. IL‐1β promotes inflammation and has been implicated in the degradation of extracellular matrix components, leading to wrinkles and sagging skin [26].

Aging is frequently associated with chronic, low‐grade inflammation, a phenomenon known as “inflammaging.” This persistent inflammation can damage skin cells and extracellular matrix components, accelerating the aging process. Inflammaging is characterized by elevated levels of inflammatory markers and cytokines, which contribute to the degradation of collagen and elastin in the skin. The continuous inflammation promotes the breakdown of these structural proteins, leading to visible signs of aging such as wrinkles and sagging skin [27].

For conditions such as psoriasis and atopic dermatitis, the inflammatory profiles are particularly relevant in the context of aging. Psoriasis is driven by a Th1/Th17‐mediated inflammatory response, involving cytokines like IL‐17 and IL‐22. These cytokines exacerbate inflammation and contribute to the chronic nature of psoriasis, which can be compounded by age‐related immune decline [28]. In contrast, atopic dermatitis is associated with a Th2‐dominant response characterized by elevated levels of IL‐4 and IL‐13. This inflammatory profile is known to interact with the aging process, leading to persistent inflammation and exacerbation of skin symptoms [29, 30].

### 3.4 Genetic Predisposition

Genetic predisposition has a major influence on an individual's aging process and skin condition. Specific genes are involved in regulating cellular repair, oxidative stress responses, and producing structural proteins such as collagen and elastin. Variations or mutations in these genes can influence how quickly the skin ages [14, 20].

Certain genetic disorders, such as progeria and Werner syndrome, provide insights into the genetic basis of aging. These disorders are characterized by accelerated aging due to mutations in specific genes involved in DNA repair and maintenance. Studying these conditions helps identify key genetic pathways that contribute to normal aging processes [4, 31].

Family history can also provide clues about genetic influences on aging. Individuals with a family history of longevity often exhibit slower aging processes, including better skin condition, suggesting that genetic factors play a significant role in the aging process [4, 13, 20].

## 4 Extrinsic Factors Affecting Skin Aging

### 4.1 UV Radiation

Ultraviolet (UV) radiation from the sun is a major external factor that contributes to skin aging, a process called photoaging. UV exposure damages skin cells by generating free radicals, which result in oxidative stress and DNA damage. It also causes direct DNA mutations, mainly through the formation of cyclobutane pyrimidine dimers (CPDs) and 6–4 photoproducts. Photoaging involves multiple mechanisms, including oxidative stress, DNA damage, inflammation, and the upregulation of MMPs [3].

UV radiation causes mutations in skin cells, resulting in the activation of oncogenes and the inactivation of tumor suppressor genes. Additionally, UV exposure increases the production of ROS, which harm cellular components like lipids, proteins, and DNA. Furthermore, UV radiation induces an inflammatory response, contributing to the breakdown of the skin's extracellular matrix. One of the critical effects of UV exposure is the upregulation of MMPs, enzymes that degrade collagen and elastin fibers, causing wrinkles and a loss of skin elasticity [3, 19].

### 4.2 Pollution

Environmental pollutants, including polycyclic aromatic hydrocarbons (PAHs), particulate matter (PM), heavy metals, and volatile organic compounds (VOCs), have detrimental effects on the skin. These pollutants penetrate the skin and generate oxidative stress, leading to inflammation and cellular damage [32].

Pollution‐induced skin aging involves several mechanisms: pollutants increase the production of ROS, leading to oxidative damage to skin cells and extracellular matrix components. Chronic exposure to pollutants induces persistent inflammation, contributing to skin aging and promotes the onset of various skin disorders. Additionally, pollutants can impair the skin barrier function, resulting in increased transepidermal water loss and reduced skin hydration [33].

### 4.3 Lifestyle Factors

Lifestyle factors significantly impact skin health and aging. A well‐balanced diet filled with antioxidants, vitamins, and minerals helps protect the skin from oxidative stress and promotes healthy aging, with nutrients like vitamins C and E, carotenoids, flavonoids, and omega‐3 fatty acids demonstrating skin‐protective properties [34]. In contrast, smoking accelerates skin aging by reducing blood flow and oxygen to the skin, depleting essential nutrients, and increasing free radical production, leading to premature wrinkles and loss of skin elasticity due to oxidative stress and collagen degradation. Similarly, excessive alcohol consumption dehydrates the skin and impairs its barrier function, causing dryness and accelerated aging, while promoting inflammation and oxidative stress, which further contribute to skin damage [35]. Additionally, adequate sleep is crucial for skin health, allowing for repair and regeneration processes, whereas poor sleep quality and duration are linked to increased signs of aging, such as dark circles, wrinkles, and reduced skin elasticity [34].

### 4.4 Skincare Practices

Skincare practices are essential in mitigating skin aging. Skincare products containing retinoids, antioxidants, peptides, and sunscreens protect against environmental damage, promote collagen production, and improve skin texture and hydration [23]. Consistent skincare routines, which include cleansing, moisturizing, and sun protection, are vital for maintaining skin health. Regular exfoliation aids in removing dead skin cells and encourages cell turnover, while adequate hydration is crucial for maintaining the skin barrier and preventing dryness [6]. Conversely, improper skincare practices, such as over‐exfoliation, using harsh products, or neglecting sun protection, can exacerbate skin aging. Products with high concentrations of irritants or allergens can lead to inflammation and damage the skin barrier [5, 11].

## 5 Molecular Mechanisms

### 5.1 Collagen and Elastin Breakdown

Collagen and elastin, essential proteins providing structural integrity and elasticity to the skin, undergo breakdown over time due to various intrinsic and extrinsic factors, resulting in the loss of skin elasticity and wrinkle formation. Intrinsic factors, such as aging‐related changes in fibroblast activity and collagen turnover, lead to decreased collagen synthesis and increased collagen breakdown [18]. Extrinsic factors, including UV radiation, pollution, and smoking, accelerate collagen and elastin breakdown by inducing oxidative stress and activating MMPs [6, 36].

### 5.2 Matrix Metalloproteinases (MMPs)

MMPs are a family of enzymes responsible for degrading extracellular matrix proteins, such as collagen and elastin. They play a crucial role in tissue remodeling and wound healing; however, they also contribute to skin aging and wrinkle formation [37]. UV radiation upregulates MMP expression in the skin, leading to increased collagen degradation and impaired skin structure. Chronic inflammation stimulates MMP production, contributing to sustained matrix degradation and skin aging. Retinoids inhibit MMP activity and promote collagen synthesis, making them effective anti‐aging treatments [36].

### 5.3 Glycation

Glycation is a non‐enzymatic process in which sugars react with proteins, resulting in the production of AGEs. Glycation affects skin proteins such as collagen and elastin, impairing their structure and function. AGEs cross‐link collagen and elastin fibers, making them stiff and less flexible, which contributes to skin stiffness and wrinkle formation. Glycation induces oxidative stress and inflammation, further exacerbating skin aging. Antioxidants and anti‐glycation agents can help mitigate the effects of glycation on skin aging [6, 38].

### 5.4 Inflammation

Chronic inflammation is a key feature of aging, contributing to skin degradation by promoting tissue damage and impairing repair mechanisms. Inflammatory cytokines and chemokines stimulate MMP production and collagen breakdown, leading to wrinkle formation and loss of skin elasticity. Infiltration of immune cells, such as macrophages and T‐cells, into the skin further exacerbates inflammation and tissue damage. Chronic inflammatory skin conditions, such as psoriasis and atopic dermatitis, accelerate skin aging and increase the risk of developing age‐related skin disorders [39].

## 6 Clinical Manifestations of Aging Skin

### 6.1 Wrinkles and Fine Lines

Wrinkles and fine lines are hallmark signs of aging skin, typically developing gradually over time. They result from a combination of intrinsic factors, such as decreased collagen and elastin production, and extrinsic factors, such as UV exposure and repetitive facial expressions. Initially, fine lines may appear around areas of facial movement, such as the eyes (crow's feet) or mouth (smile lines). With continued aging and skin damage, wrinkles deepen and become more pronounced, affecting the overall texture and appearance of the skin [18, 40].

### 6.2 Loss of Firmness and Elasticity

Loss of firmness and elasticity are primarily attributed to the degradation of collagen and elastin fibers in the skin. Collagen provides structural support and firmness, while elastin contributes to skin elasticity and resilience. With age, the production of these proteins declines, leading to sagging skin and a loss of youthful contours. Factors such as UV exposure, smoking, and poor skincare exacerbate this process, resulting in visibly lax and drooping skin, especially in areas prone to gravitational effects, such as the cheeks and jawline [3, 6, 41].

### 6.3 Pigmentation Changes

Pigmentation changes are common signs of aging skin, appearing in various forms. Age spots, or solar lentigines, are flat, brown spots that typically develop on sun‐exposed areas like the face, hands, and arms. These spots arise due to the localized accumulation of melanin caused by long‐term UV exposure. Individuals with lighter skin tones are particularly susceptible to solar lentigines, as their skin is more prone to UV‐induced damage [41].

Melasma, on the other hand, characterized by irregular patches of darkened skin, often occurs on the face and is particularly prevalent in women with hormonal fluctuations and sun exposure. This condition results from both hormonal influences and UV radiation. Although melasma can occur in all skin types, it is more common in individuals with darker skin tones (Fitzpatrick types III–VI), who experience a greater disease burden due to their higher melanin content and genetic predisposition to hyperpigmentation [42]. Additionally, post‐inflammatory hyperpigmentation (PIH) is a significant concern for patients with the skin of color. This condition arises after skin injury or inflammation, resulting in dark spots that can persist for long periods. Patients with Fitzpatrick skin types IV–VI experience higher rates of PIH, and the condition often leaves lasting pigmentation changes, contributing to an uneven complexion and exacerbating the appearance of aging skin [43].

These pigmentary disorders, along with uneven skin tone and freckles, disproportionately affect individuals with the skin of color and can significantly impact their quality of life. The chronic nature and psychosocial effects of pigmentation changes in darker‐skinned individuals warrant further discussion to address this population's unique challenges [42, 43].

### 6.4 Dryness and Texture Changes

Skin dryness and texture changes are common features of aging skin, resulting from both extrinsic and intrinsic factors. Intrinsic factors, such as reduced sebum production and compromised skin barrier function, lead to dryness and rough texture. With age, a decrease in lipid content leads to increased transepidermal water loss and diminished moisture retention. Extrinsic factors, including low humidity, harsh weather conditions, and exposure to irritants, further exacerbate dryness and compromise skin texture. Additionally, cumulative UV damage and inflammation disrupt skin cell turnover, leading to a rough, uneven complexion [6, 22, 43].

## 7 Assessment and Measurement of Skin Aging

### 7.1 Clinical Evaluation

Clinical evaluation of skin aging employs various techniques to assess and quantify age‐related changes. Dermatologists and skincare professionals perform visual inspections to identify aging indicators, including wrinkles, pigmentation changes, and reduced elasticity. Skin texture analysis devices provide quantitative measurements of skin roughness, smoothness, and surface irregularities. Elasticity assessment tools, like the Cutometer, measure skin elasticity by evaluating its ability to recoil after deformation. Additionally, standardized scales such as the Glogau scale and the Fitzpatrick wrinkle scale are used to grade the severity of wrinkles and fine lines based on their depth and distribution [44, 45].

### 7.2 Biomarkers

Biomarkers are measurable indicators of biological processes associated with aging, providing valuable insights into the mechanisms and progression of skin aging in research and clinical practice. Genetic biomarkers, including variations associated with collagen synthesis, antioxidant defense, and inflammation, indicate susceptibility to skin aging. Protein biomarkers, such as MMPs, cytokines, and growth factors, reflect changes in extracellular matrix remodeling, inflammation, and tissue repair. Additionally, epigenetic biomarkers, including DNA methylation patterns, histone modifications, and microRNA expression profiles, serve as indicators of chronological and photoaging [45, 46, 47].

### 7.3 Imaging Technologies

Advanced imaging technologies enable non‐invasive assessment and monitoring of skin aging at the cellular and molecular levels. High‐resolution photography techniques, such as dermoscopy and confocal microscopy, provide detailed visualization of skin structures and pathological changes, allowing for precise diagnosis and monitoring of aging‐related alterations. Optical coherence tomography (OCT) employs light waves to produce cross‐sectional images of the skin. This technique allows for the visualization of skin layers and the measurement of both epidermal and dermal thicknesses, as well as collagen density. Multiphoton microscopy allows for label‐free imaging of skin components, such as collagen fibers, elastin, and melanin, with high resolution and depth penetration, facilitating the study of skin aging mechanisms in vivo [48, 49, 50, 51, 52].

## 8 Interventions and Prevention Strategies

### 8.1 Topical Treatments

Retinoids, which are derivatives of vitamin A like tretinoin, retinol, and adapalene, stimulate collagen synthesis, increase skin cell turnover, and improve the overall skin texture and tone. Retinoids bind to retinoic acid receptors in the skin, leading to increased epidermal cell proliferation and reduced breakdown of collagen, which is crucial for maintaining skin elasticity. Despite their efficacy, retinoids can cause side effects such as irritation, dryness, peeling, and increased photosensitivity, especially during the initial phases of treatment. Due to these side effects, retinoids are often contraindicated for individuals with sensitive skin or conditions such as rosacea and eczema. Pregnant or breastfeeding women are also advised to avoid retinoid use due to the risk of teratogenicity [53, 54].

Additionally, antioxidants such as vitamins C and E, coenzyme Q10, and green tea extract neutralize free radicals, reduce oxidative stress, and protect against UV‐induced damage, which accelerates collagen breakdown and contributes to the formation of wrinkles and age spots. Vitamin C, for instance, plays a dual role as both an antioxidant and a stimulator of collagen production, making it a popular ingredient in anti‐aging formulations. However, antioxidant‐based treatments typically require consistent, long‐term use to produce visible benefits and may be less effective in more advanced cases of photoaging [55].

Peptide‐based formulations are another class of topicals that have gained attention for their role in stimulating collagen production, improving skin elasticity, and reducing the appearance of fine lines and wrinkles. Peptides, such as palmitoyl pentapeptide‐4, mimic growth factors that naturally decline with age and help to reinforce the skin's structural integrity. Peptides are generally well‐tolerated with minimal side effects, making them suitable for most skin types, including sensitive skin [53, 55].

While each of these topical treatments provides valuable benefits, they also come with limitations. Retinoids, while effective, may not be suitable for individuals with highly sensitive or reactive skin. Antioxidants, although protective, require long‐term use for noticeable results, and peptides, while less irritating, may offer more modest improvements compared to other interventions. For optimal results, topical treatments are often combined with other anti‐aging therapies, such as laser treatments or chemical peels, to achieve a more comprehensive improvement in skin appearance [54, 55].

### 8.2 Procedures

Laser resurfacing with fractional laser treatments targets specific skin layers to stimulate collagen production, improve skin texture, and reduce wrinkles and pigmentation. These treatments worked by delivering controlled heat to the dermis, encouraging collagen remodeling, and improving overall skin elasticity. Typically, multiple sessions were required, with visible improvements occurring over several months as new collagen formed [56].

Additionally, chemical peels using solutions like glycolic acid, salicylic acid, and trichloroacetic acid (TCA) exfoliate the skin, promote cell turnover, and enhance skin tone and texture. The depth of penetration varied depending on the chemical solution used. Superficial peels required fewer sessions, while deeper peels not only offered more significant results but also had longer recovery times. Patients often saw improvements in pigmentation and fine lines within weeks of treatment [57]. Moreover, microdermabrasion techniques, including diamond‐tip or crystal microdermabrasion, mechanically exfoliate dead skin cells, stimulate cell renewal, and boost skin radiance. This minimally invasive procedure required little downtime and was often performed as a series of treatments for optimal results. Patients could expect immediate improvements in skin texture, though deeper benefits became apparent over multiple sessions [58].

Finally, photodynamic therapy (PDT) is a non‐invasive option for addressing signs of aging. PDT worked by applying a photosensitizing agent to the skin and activating it with a specific wavelength of light. This therapy reduced sun damage, wrinkles, and precancerous lesions by selectively targeting damaged skin cells while sparing healthy tissues. Results typically unfolded over several weeks and required multiple sessions depending on the severity of skin damage [59].

### 8.3 Lifestyle Modifications

A balanced diet that includes antioxidants, vitamins, minerals, and omega‐3 fatty acids promotes skin health by reducing inflammation and oxidative stress [34], while regular exercise boosts blood circulation, stimulates collagen production, and improves skin elasticity and firmness [60]. Additionally, daily sunscreen use, gentle cleansing, moisturizing, and the consistent use of topical treatments tailored to individual skin concerns are essential for maintaining skin health and preventing premature aging [23].

### 8.4 Emerging Therapies

Stem cell therapy uses stem cell–derived products and treatments to leverage the regenerative potential of stem cells for repairing and rejuvenating aging skin [61]. Platelet‐rich plasma (PRP) therapy employs growth factors and cytokines from the patient's own blood to stimulate collagen production, enhance skin texture, and improve overall skin quality [62]. Additionally, nanotechnology utilizes nanoparticle‐based delivery systems to enhance the efficacy and penetration of skincare ingredients, allowing for targeted delivery and improved anti‐aging effects [63].

## 9 Future Directions and Research Gaps

Advancements in genomics and proteomics have paved the way for identifying genetic markers and protein signatures associated with skin aging. These discoveries offer valuable insights into the underlying mechanisms and potential therapeutic targets for anti‐aging interventions. However, knowledge gaps remain, particularly regarding the long‐term effects of emerging therapies, such as stem cell–based and nanotechnology‐based treatments. While promising, these interventions require further investigation to ensure their safety and efficacy over extended periods.

One potential innovation lies in the development of precision medicine approaches. Leveraging advancements in omics technologies and machine learning algorithms, personalized anti‐aging interventions can be tailored to individual genetic predispositions, lifestyle factors, and skin characteristics. Additionally, research in epigenetics highlights how environmental factors and lifestyle choices influence gene expression, contributing to skin aging. This opens the door for more personalized interventions targeting these pathways.

Despite progress, there is still a significant lack of diversity in skin aging research, as most studies focus on Caucasian populations. This creates a gap in understanding how aging affects different ethnic groups, including differences in aging patterns, risk factors, and responses to treatment. More diverse studies are critical to addressing these disparities.

Nanomedicine continues to show potential as an anti‐aging tool, with the development of nanotechnology‐based delivery systems improving the targeted delivery of compounds. These systems enhance bioavailability and efficacy while minimizing side effects. Concurrently, ongoing microbiome research has begun to reveal the skin microbiome's role in maintaining skin health and its potential implications for age‐related skin changes. Future therapies may increasingly target the microbiome to maintain youthful skin.

There is still much to learn about intrinsic aging processes, including cellular senescence and telomere shortening. While extrinsic factors like UV exposure are well documented, intrinsic factors deserve deeper exploration. In this context, CRISPR‐based therapies may offer innovative solutions by correcting genetic mutations that contribute to premature aging disorders and other age‐related skin conditions.

Lastly, tissue engineering and regenerative medicine present exciting opportunities. Techniques like 3D bioprinting and stem cell–based therapies could regenerate aged or damaged skin, restoring youthful properties. Moreover, advances in biomedical imaging, such as multiphoton microscopy and OCT, provide new ways to visualize skin aging processes at a cellular and molecular level, facilitating early detection and monitoring [51, 52, 63, 64, 65, 66, 67, 68].

## 10 Conclusion

This review has provided a comprehensive overview of the factors influencing skin and intrinsic aging. We explored the intricate interplay of genetic predisposition, hormonal changes, metabolic processes, environmental exposures, and lifestyle factors in shaping the aging process. From cellular senescence and oxidative stress to UV radiation and lifestyle habits, numerous internal and external factors contribute to the development of age‐related changes in the skin. Understanding the mechanisms underlying skin aging has significant implications for healthcare and skincare practices. By embracing interdisciplinary approaches and leveraging cutting‐edge technologies, we can pave the way for transformative advancements in anti‐aging medicine and skincare, ultimately enhancing the quality of life for individuals worldwide.

## Author Contributions

The authors made substantial contributions to the study's conception and design, data acquisition, and analysis, as well as the interpretation of results. They were actively involved in drafting the manuscript, ensuring it contained significant intellectual content. Furthermore, they agreed to submit the article to the current journal, approved the final version for publication, and assumed responsibility for the entire work.

## Ethics Statement

The authors have nothing to report.

## Conflicts of Interest

The authors declare no conflicts of interest.

## Data Availability

The data that support the findings of this study are available from the corresponding author upon reasonable request.

## References

[1] L. Hayflick , “The Future of Ageing,” Nature 408, no. 6809 (2000): 267–269. 11089985 10.1038/35041709

[2] T. B. L. Kirkwood , “Understanding the Odd Science of Aging,” Cell 120, no. 4 (2005): 437–447. 15734677 10.1016/j.cell.2005.01.027

[3] B. A. Gilchrest , “Photoaging,” Journal of Investigative Dermatology 133, no. E1 (2013): E2–E6. 10.1038/skinbio.2013.176 23820721

[4] M. R. Schneider and H. Mitsui , “The Relationship Between Skin and Systemic Aging,” Dermatology Clinics 36, no. 1 (2018): 81–90.

[5] M. Yaar and B. A. Gilchrest , “Photoageing: Mechanism, Prevention and Therapy,” British Journal of Dermatology 157, no. 5 (2007): 874–887. 17711532 10.1111/j.1365-2133.2007.08108.x

[6] M. A. Farage , K. W. Miller , P. Elsner , and H. I. Maibach , “Intrinsic and Extrinsic Factors in Skin Ageing: A Review,” International Journal of Cosmetic Science 30, no. 2 (2008): 87–95. 18377617 10.1111/j.1468-2494.2007.00415.x

[7] A. J. Scott , M. Ellison , and D. A. Sinclair , “The Economic Value of Targeting Aging,” Nature Aging 1 (2021): 616–623. 37117804 10.1038/s43587-021-00080-0 PMC10154220

[8] V. S. Mak , “Technologies and Dietary Change: The Pharmaceutical Nexus and the Marketing of Anti‐Aging Functional Food in a Chinese Society,” Food and Foodways 29, no. 4 (2021): 309–330.

[9] J. S. Mandelblatt , M. H. Antoni , T. N. Bethea , et al., “Gerotherapeutics: Aging Mechanism‐Based Pharmaceutical and Behavioral Interventions to Reduce Cancer Racial and Ethnic Disparities,” Journal of the National Cancer Institute djae211 (2024): 1730–1738. 10.1093/jnci/djae211 PMC11884862 39196709

[10] C. C. Zouboulis , E. Makrantonaki , and N. C. Nicolaides , “Hormonal Therapy in Intrinsic Skin Aging,” Clinical Interventions in Aging 9 (2014): 1153–1159.25083133

[11] L. Rittie and G. J. Fisher , “Natural and Sun‐Induced Aging of Human Skin,” Cold Spring Harbor Perspectives in Medicine 5, no. 1 (2015): a015370. 25561721 10.1101/cshperspect.a015370 PMC4292080

[12] J. Campisi and F. d'Adda di Fagagna , “Cellular Senescence: When Bad Things Happen to Good Cells,” Nature Reviews Molecular Cell Biology 8, no. 9 (2007): 729–740. 17667954 10.1038/nrm2233

[13] B. G. Childs , M. Durik , D. J. Baker , and J. M. van Deursen , “Cellular Senescence in Aging and Age‐Related Disease: From Mechanisms to Therapy,” Nature Medicine 21, no. 12 (2015): 1424–1435. 10.1038/nm.4000 PMC4748967 26646499

[14] J. H. Chen , C. N. Hales , and S. E. Ozanne , “DNA Damage, Cellular Senescence and Organismal Ageing: Causal or Correlative?,” Nucleic Acids Research 35, no. 22 (2007): 7417–7428. 17913751 10.1093/nar/gkm681 PMC2190714

[15] M. A. Blasco , “Telomere Length, Stem Cells and Aging,” Nature Chemical Biology 3, no. 10 (2007): 640–649. 17876321 10.1038/nchembio.2007.38

[16] S. Kawanishi and S. Oikawa , “Mechanism of Telomere Shortening by Oxidative Stress,” Annals of the New York Academy of Sciences 1019, no. 1 (2004): 278–284. 15247029 10.1196/annals.1297.047

[17] T. Finkel and N. J. Holbrook , “Oxidants, Oxidative Stress and the Biology of Ageing,” Nature 408, no. 6809 (2000): 239–247. 11089981 10.1038/35041687

[18] G. J. Fisher , J. Varani , and J. J. Voorhees , “Looking Older: Fibroblast Collapse and Therapeutic Implications,” Archives of Dermatology 144, no. 5 (2008): 666–672. 18490597 10.1001/archderm.144.5.666 PMC2887041

[19] R. E. B. Watson , N. K. Gibbs , C. E. M. Griffiths , and M. J. Sherratt , “Damage to Skin Extracellular Matrix Induced by UV Exposure,” Antioxidants & Redox Signaling 21, no. 7 (2014): 1063–1077. 24124905 10.1089/ars.2013.5653

[20] C. López‐Otín , M. A. Blasco , L. Partridge , M. Serrano , and G. Kroemer , “The Hallmarks of Aging,” Cell 153, no. 6 (2013): 1194–1217. 23746838 10.1016/j.cell.2013.05.039 PMC3836174

[21] S. Verdier‐Sévrain and F. Bonte , “Skin Hydration: A Review on Its Molecular Mechanisms,” Journal of Cosmetic Dermatology 6, no. 2 (2007): 75–82. 17524122 10.1111/j.1473-2165.2007.00300.x

[22] R. Ganceviciene , A. I. Liakou , A. Theodoridis , E. Makrantonaki , and C. C. Zouboulis , “Skin anti‐aging strategies,” Dermato‐Endocrinology 4, no. 3 (2012): 308–319. 23467476 10.4161/derm.22804 PMC3583892

[23] L. Baumann , “Skin Ageing and Its Treatment,” Journal of Pathology 211, no. 2 (2007): 241–251. 17200942 10.1002/path.2098

[24] N. Ahmed , “Advanced Glycation Endproducts—Role in Pathology of Diabetic Complications,” Diabetes Research and Clinical Practice 67, no. 1 (2005): 3–21. 15620429 10.1016/j.diabres.2004.09.004

[25] E. L. Goldberg and V. D. Dixit , “Drivers of Age‐Related Inflammation and Strategies for Healthspan Extension,” Immunological Reviews 265, no. 1 (2015): 63–74. 25879284 10.1111/imr.12295 PMC4400872

[26] R. Thomas , W. Wang , and D.‐M. Su , “Contributions of Age‐Related Thymic Involution to Immunosenescence and Inflammaging,” Immunity & Ageing 17 (2020): 2, 10.1186/s12979-020-0173-8. 31988649 PMC6971920

[27] A. M. Weber and C. M. Niessen , “Pro‐Inflammatory Cytokines and Immunity in Skin Homeostasis,” Current Opinion in Pharmacology 12, no. 4 (2012): 453–461.

[28] E. Fitsiou , T. Pulido , J. Campisi , F. Alimirah , and M. Demaria , “Cellular Senescence and the Senescence‐Associated Secretory Phenotype as Drivers of Skin Photoaging,” Journal of Investigative Dermatology 141, no. 4S (2021): 1119–1126, 10.1016/j.jid.2020.09.031. 33349436

[29] G. S. Bocheva , R. M. Slominski , and A. T. Slominski , “Immunological Aspects of Skin Aging in Atopic Dermatitis,” International Journal of Molecular Sciences 22 (2021): 5729, 10.3390/ijms22115729. 34072076 PMC8198400

[30] L. Zhou , A. Leonard , A. B. Pavel , et al., “Age‐Specific Changes in the Molecular Phenotype of Patients With ModerateTo‐Severe Atopic Dermatitis,” Journal of Allergy and Clinical Immunology 144 (2019): 144–156, 10.1016/j.jaci.2019.01.015. 30685456

[31] T. B. L. Kirkwood and S. Melov , “On the Programmed/Non‐programmed Nature of Ageing Within the Life History,” Current Biology 21, no. 18 (2011): R701–R707. 21959160 10.1016/j.cub.2011.07.020

[32] A. Vierkötter and J. Krutmann , “Environmental Influences on Skin Aging and Ethnic‐Specific Manifestations,” Dermato‐Endocrinology 4, no. 3 (2012): 227–231. 23467702 10.4161/derm.19858 PMC3583881

[33] J. Krutmann , D. Moyal , W. Liu , et al., “Pollution and Skin: From Epidemiological and Mechanistic Studies to Clinical Implications,” Journal of Dermatological Science 76, no. 3 (2014): 163–168. 25278222 10.1016/j.jdermsci.2014.08.008

[34] E. Makrantonaki and C. C. Zouboulis , “The Skin as a Mirror of the Aging Process in the Human Organism—State of the Art and Results of the Aging Research in the German National Genome Research Network 2 (NGFN‐2),” Experimental Gerontology 42, no. 9 (2007): 879–886. 17689905 10.1016/j.exger.2007.07.002

[35] J. H. Chung , V. N. Hanft , and S. Kang , “Aging and photoaging,” Journal of the American Academy of Dermatology 49, no. 4 (2003): 690–697. 14512918 10.1067/s0190-9622(03)02127-3

[36] T. Quan , Z. Qin , W. Xia , Y. Shao , J. J. Voorhees , and G. J. Fisher , “Mechanisms of UV‐Induced Skin Aging,” Journal of Investigative Dermatology 133 (2013): 1–6.

[37] T. Quan , Z. Qin , W. Xia , Y. Shao , J. J. Voorhees , and G. J. Fisher , “Matrix‐Degrading Metalloproteinases in Photoaging,” Journal of Investigative Dermatology Symposium Proceedings 14, no. 1 (2010): 20–24. 10.1038/jidsymp.2009.8 PMC2909639 19675548

[38] F. Rijken , P. L. Bruijnzeel , H. van Weelden , et al., “The Glycation Pattern of Aged Collagen in Human Skin and its Relationship to Yellowing,” Journal of Investigative Dermatology 127 (2007): 2646–2654.

[39] L. Chen , Y. Hu , W. Wang , et al., “Chronic Inflammation and Skin Aging,” Journal of Dermatology 39 (2012): 751–759.22924439

[40] G. J. Fisher , Z. Q. Wang , S. C. Datta , et al., “Pathophysiology of Premature Skin Aging Induced by Ultraviolet Light,” New England Journal of Medicine 347 (2002): 1419–1428. 9358139 10.1056/NEJM199711133372003

[41] J. L. Bolognia , J. V. Schaffer , L. Cerroni , et al., “Aging Skin,” American Journal of Clinical Dermatology 13 (2012): 435–448.

[42] P. E. Grimes , “Melasma: Etiologic and Therapeutic Considerations,” Archives of Dermatology 145, no. 4 (2009): 386–392. 10.1001/archderm.131.12.1453 7492140

[43] F. Flament , R. Bazin , S. Laquieze , V. Rubert , E. Simonpietri , and B. Piot , “Effect of the Sun on Visible Clinical Signs of Aging in Caucasian Skin,” Clinical, Cosmetic and Investigational Dermatology 6 (2013): 221–232. 24101874 10.2147/CCID.S44686 PMC3790843

[44] B. A. Gilchrest , J. Krutmann , Y. Takema , et al., “Skin Aging and its Prevention,” Dermatologic Clinics 31 (2013): 87–95.

[45] M. A. Farage , H. I. Maibach , R. H. Nachtigall , et al., “Skin Measurements and Skin Physiology: Their Role in Understanding Skin Function and Efficacy of Topical and Systemic Therapies,” Dermatologic Clinics 31 (2013): 21–26.23159173

[46] J. Bhawan , “Histopathology of Photoaged Skin,” Dermatology Clinics 27, no. 4 (2009): 401–411.

[47] A. S. Wang and O. Dreesen , “Biomarkers of Cellular Senescence and Skin Aging,” Frontiers in Genetics 9 (2018): 247. 30190724 10.3389/fgene.2018.00247 PMC6115505

[48] B. Querleux , T. Baldeweck , M. Dandurand , et al., “New Insights Into the Characterization of Skin Aging by In Vivo Reflectance Confocal Microscopy,” Dermatology 218 (2009): 3–8.

[49] G. Leung , L. Beck , P. Herman , et al., “Non‐Invasive Imaging of Skin Physiology and Percutaneous Penetration Using Fluorescence Spectral and Lifetime Imaging With Multiphoton and Confocal Microscopy,” European Journal of Dermatology 21 (2011): 43–53. 10.1016/j.ejpb.2010.12.023 21256962

[50] S. Cho , M. H. Shin , Y. K. Kim , et al., “Non‐Invasive Assessment of the Depth Profile of Epidermal Melanin and Dermal Haemoglobin by Reflectance Confocal Microscopy,” British Journal of Dermatology 156 (2007): 85–92.17199572

[51] K. König , H. G. Breunig , I. Riemann , et al., “Clinical Multiphoton Tomography,” Journal of Biophotonics 12 (2019): e201800214. 10.1002/jbio.200710022 19343631

[52] J. H. Park , J. Lee , C. H. Kim , et al., “Optical Coherence Tomography in Dermatology: Technical and Clinical Aspects,” Journal of Dermatological Science 91 (2018): 121–127.

[53] M. El‐Domyati , S. Attia , F. Saleh , et al., “Evaluation of the Effects of Topical Tretinoin (Retinoic Acid) on Photoaged Skin: A Quantitative Study,” Journal of the American Academy of Dermatology 47 (2002): 780–787.12399776

[54] S. Mukherjee , A. Date , V. Patravale , H. C. Korting , A. Roeder , and G. Weindl , “Retinoids in the Treatment of Skin Aging: An Overview of Clinical Efficacy and Safety,” Clinical Interventions in Aging 1, no. 4 (2006): 327–348. 18046911 10.2147/ciia.2006.1.4.327 PMC2699641

[55] P. K. Farris , “Topical Vitamin C: A Useful Agent for Treating Photoaging and Other Dermatologic Conditions,” Dermatologic Surgery 31, no. 7 Pt 2 (2005): 814–818. 16029672 10.1111/j.1524-4725.2005.31725

[56] M. R. Alexiades‐Armenakas , J. S. Dover , and K. A. Arndt , “The Spectrum of Laser Skin Resurfacing: Nonablative, Fractional, and Ablative Laser Resurfacing,” Journal of the American Academy of Dermatology 58, no. 5 (2008): 719–737. 18423256 10.1016/j.jaad.2008.01.003

[57] K. C. Lee , M. R. Roh , J. H. Choi , et al., “Chemical Peels,” Facial Plastic Surgery Clinics of North America 14 (2006): 127–138.

[58] K. L. Wells and R. A. Oberhelman , “Microdermabrasion in Aesthetic Dermatology,” Facial Plastic Surgery Clinics of North America 15, no. 1 (2007): 55–61.17317556

[59] E. Kohl , M. Koller , F. Zeman , et al., “Daylight Photodynamic Therapy Versus Cryosurgery for the Treatment and Prophylaxis of Actinic Keratoses of the Face: Protocol of a Multicenter, Prospective, Randomized, Controlled, Two‐Armed Study,” BMC Dermatology 17, no. 1 (2017): 12. 29070025 10.1186/s12895-017-0064-7 PMC5657041

[60] J. E. Kim , J. Lee , Y. Park , et al., “Exercise‐Induced Oxidative Stress Responses in the Pediatric Population,” Frontiers in Bioscience (Elite Edition) 1 (2009): 478–486.

[61] W. S. Kim , B. S. Park , J. H. Sung , et al., “The Wound‐Healing and Antioxidant Effects of Adipose‐Derived Stem Cells,” Expert Opinion on Biological Therapy 9 (2008): 879–887. 10.1517/14712590903039684 19522555

[62] M. Alam , J. S. Dover , K. A. Arndt , et al., “Platelet‐Rich Plasma: A Review,” Facial Plastic Surgery 25 (2009): 334–342.

[63] H. S. Yoo , J. Kim , K. Jung , et al., “Nanoencapsulation of Retinol Improves its Photostability and Bioavailability,” International Journal of Pharmaceutics 403 (2011): 170–176.20934495

[64] S. Zhang , H. Gao , Y. Li , et al., “Genomics of Skin Aging,” Ageing Research Reviews 55 (2019): 100956.31479764

[65] B. Dréno , E. Araviiskaia , E. Berardesca , et al., “Microbiome in Healthy Skin, Update for Dermatologists,” Journal of the European Academy of Dermatology and Venereology 30, no. 12 (2016): 2038–2047. 27735094 10.1111/jdv.13965 PMC6084363

[66] Y. C. Kim , K. Park , H. J. Lee , et al., “Nanotechnology‐Based Cosmeceuticals: Innovation, Trends, and Future Perspectives,” Journal of Cosmetic Dermatology 18 (2019): 366–371.

[67] F. Guo , Z. Zhang , R. Chen , et al., “CRISPR‐Based Therapeutics for Skin Disorders,” Human Genetics 138 (2019): 721–726.

[68] B. M. Hariri , S. J. Park , H. P. Nguyen , et al., “Precision Medicine in Skin Care: An Emerging Approach for Targeted Anti‐Aging Therapy,” Journal of Drugs in Dermatology 19 (2020): 24–27.

